# Induction of Spermiation in Sterlet *Acipenser ruthenus* by PLGA Microparticle Delivery with Sustained Alarelin Release

**DOI:** 10.3390/ani11113305

**Published:** 2021-11-19

**Authors:** Peter Podhorec, Jindřiška Knowles, Jakub Vysloužil, Sergii Boryshpolets, Kateřina Kubová, Marek Rodina, Vitaliy Kholodnyy, Anatolii Sotnikov, David Gela, Borys Dzyuba

**Affiliations:** 1 South Bohemian Research Center of Aquaculture and Biodiversity of Hydrocenoses, Faculty of Fisheries and Protection of Waters, University of South Bohemia in České Budějovice, Zátiší 728/II, 389 25 Vodňany, Czech Republic; matejkovaj@frov.jcu.cz (J.K.); sboryshpolets@frov.jcu.cz (S.B.); rodina@frov.jcu.cz (M.R.); vkholodnyy@frov.jcu.cz (V.K.); asotnikov@frov.jcu.cz (A.S.); gela@frov.jcu.cz (D.G.); bdzyuba@frov.jcu.cz (B.D.); 2 Department of Pharmaceutical Technology, Faculty of Pharmacy, Masaryk University, Palackeho trida 1946/1, 612 00 Brno, Czech Republic; jakub.vyslouzil@gmail.com (J.V.); kubovak@pharm.muni.cz (K.K.)

**Keywords:** sperm, reproduction, sturgeon, sustained release

## Abstract

**Simple Summary:**

Cultured sterlet *Acipenser ruthenus* males do not usually undergo spontaneous spermiation, and if any sperm is obtained without hormone treatment, it is generally of diminished quality. We compared efficacy of stimulation of spermiation with carp pituitary extract to that of 35 µg kg^−1^ or 200 µg kg^−1^ body weight gonadotropin-releasing hormone analogue in a sustained release system. Hormone treatments caused a significant increase in testosterone and 11-ketotestosterone, as well as induced spermiation. The delivery system based on poly (lactic-co-glycolic acid) microparticles with slow release of Alarelin at 35 µg kg^−1^ BW effectively induced spermiation, and was associated with extended sperm collection compared to carp pituitary treatment. The sustained delivery system offers an excellent option for spermiation induction in cultured sterlet, and possibly other sturgeon.

**Abstract:**

Carp pituitary treatment versus poly (lactiac-co-glycolic acid) microparticles with slow release of Alarelin at 35 µg kg^−1^ or 200 µg kg^−1^ body weight to induce spermiation was compared in sterlet *Acipenser ruthenus*. All hormone treatments initially increased testosterone and 11-ketotestosterone, with a subsequent decline in testosterone but consistent high levels of 11-ketotestosterone at 48 and 72 h post-treatment. Spermiation did not differ between hormone-treated groups, and was not detected in controls receiving saline solution. Administration of the carp pituitary led to maximum sperm production 24 h post-treatment, followed by a decrease at 48 h post-treatment, with no sperm obtained at 72 h. The effect of Alarelin at 35 µg kg^−1^ bw and carp pituitary did not differ at 24 and 48 h post-treatment, whereas 200 µg kg^−1^ bw Alarelin was associated with significantly lower spermatozoon concentration 24 h post-treatment compared to carp pituitary, with no difference in milt volume. Higher relative sperm production was observed 48 h after injection of Alarelin at 200 µg kg^−1^ bw compared to carp pituitary. Spermatozoon motility was significantly higher in fish receiving Alarelin at 35 µg kg^−1^ bw than 200 µg kg^−1^ bw. The treatment with optimal effect on inducing spermiation was poly (lactic-co-glycolic acid) microparticles with slow release of Alarelin at 35 µg kg^−1^ bw.

## 1. Introduction

The order Acipenseriformes comprises 27 species, with natural distribution in Eurasia and North America [1]. The population of all sturgeon species has declined drastically due to over-fishing, pollution, and river modifications [2], with all sturgeon species listed under Appendix II of the Convention on International Trade in Endangered Species of Wild Fauna and Flora (CITES) since 1998.

Successful completion of the life cycle of sturgeon in captivity and optimization of production techniques is essential, considering the high demand for viable fingerlings and caviar. The conditions on fish farms differ dramatically from those that broodfish are exposed to in natural habitats. Artificial environments lacking natural spawning stimuli do not induce appropriate endogenous responses from the fish [3], and cultured sturgeon often manifest reproductive dysfunction at the final level of gametogenesis [4].

Traditionally, the dysfunction is overcome by the injection of gonadotropins [carp pituitary suspension (CP), chorionic gonadotropins], to supplement production of endogenous luteinizing hormone (LH) [5]. The discovery of gonadotropin-releasing hormone (GnRH) [6] and its effects introduced a new tool for regulating reproduction in cultured fish [7]. Subsequent production of GnRH analogues (GnRHa) with modified amino acid positions has resulted in multiple increase of GnRHa effectiveness in stimulating LH secretion and ovulation [8].

Most research into male sturgeon reproduction has compared the potency of hormonally active substances dissolved in saline solution in inducing spermiation administered by intramuscular or intraperitoneal injection [9,10]. A single study of efficacy of a sustained release system in inducing spermiation in sturgeon showed negative results [4]. Sustained release of GnRHa is based on prolonged stimulation of pituitary gonadotrophs, leading to elevated secretion of LH into the bloodstream with appropriately stimulated steroidogenesis and improved spermiation [11]. Several sustained release systems are used in aquaculture: ethylene-vinyl acetate copolymer (EVAc) implants [12], solid implantable cholesterol pellets [13], oil emulsion [14], and poly (lactic-co-glycolic acid) microparticles (PLGA) [15]. Long-term sustained release systems have significantly improved sperm production in several marine fish species [16,17]. Their biocompatibility, biodegradability, and potential for administering a precise dose of liquid suspension make PLGA microparticles ideal for encapsulation of peptides like GnRHa [18].

We chose the sterlet Acipenser ruthenus as a model species for this study since, in addition to its small size compared to much bigger anadromous sturgeon and freshwater life history, males reach sexual maturity at three years [9]. Without treatment, sterlet males in captivity, as with most sturgeons, will produce no sperm, or will produce sperm of a lower quality [19].

The goal of the study was to determine the efficacy of a PLGA microparticle delivery system with sustained Alarelin release to induce spermiation in sterlet. Comparison with the most common treatment, carp pituitary suspension, was based on qualitative and quantitative parameters of obtained sperm, supported by stimulated levels of the main androgens.

## 2. Materials and Methods

### 2.1. Fish Rearing and Pre-Spawning Water Conditions

Individually tagged five to six-year-old male sterlet were held in aquaculture ponds till January. In January, 40 males of similar body weight 1.58 ± 0.24 kg (mean ± SD, one-way ANOVA, *p* = 0.59) were transferred to an indoor recirculation aquaculture system (water temperature 2 °C), and randomly divided into four groups. Each group (*n* = 10) was placed in a separate 0.8 m^3^ tank with a heater and aeration, ensuring optimal dissolved oxygen concentrations (higher than 95% of saturation).

Temperature was set to 5 °C for ten days, and then increased to 14 °C within six days (~+2 °C per day), with a further increase in temperature over the next six days to 15 °C. After 24 h, the experimental treatments were administered.

### 2.2. Treatment

#### 2.2.1. Synthesis of PLGA Microparticles

The microparticles were prepared by solvent evaporation from a multiple emulsion with Alarelin (APExBIO, Houston, TX, USA). Alarelin is a synthetic polypeptide which acts as gonadotropin-releasing hormone analogue agonist and is highly soluble in water. Resomer 753, a copolymer of lactic acid and glycolic acid (75% polylactic acid and 25% polyglycolic acid), was used as carrier.

Precisely 800 mg of PLGA of the Resomer (Evonik, Darmstadt, Germany) was weighed in a wide-necked tube, and 5 g dichloromethane (Penta, Prague, Czech Republic) was added. The contents were capped and allowed to dissolve. Meanwhile, a 10% gelatin solution (Sigma Aldrich, St. Louis, MO, USA) and 12 g of a 1% polyvinyl alcohol solution (PVA; Sigma Aldrich, USA) was heated in a water bath. Further, 10 mg of Alarelin was weighed into a microcentrifuge tube, 1.5 mL gelatin was added, and vortexed to dissolve the drug. The resulting solution was poured into the wide-necked tube containing PLGA dissolved in dichloromethane, and vortexed again to ensure emulsification. The contents of the tube were homogenized to produce a fine emulsion. Subsequent homogenization with 12 g of 1% PVA solution (T25 basic, IKA-Werke, Staufen, Germany) produced a concentrated water/oil/water emulsion, which was then diluted in 200 mL of 0.1% PVA solution containing 2% NaCl and placed under a shaft stirrer set at 450 rpm. The contents of the wide-mouth tube were poured into the external aqueous phase, and the dichloromethane was evaporated for 2 h. The resulting micro-suspension was filtered through a 250 μm screen for the separation of possible agglomerates. Isolation of the microparticles was then performed by centrifugation at 6000× *g* for 2 min. Excess water was decanted, and the microparticles were collected, stored in a freezer, and subsequently dried by lyophilization.

The content of Alarelin in PLGA microparticles was determined by high-performance liquid chromatography (HPLC). First, the microparticles were dissolved in acetone, and the resulting solution was mixed 1:1 (*v*/*v*) with a phosphate buffer of pH 7.0. The resulting mixture was filtered through a 0.45 μm membrane filter. The mixture was quantified by HPLC (Agilent 1100; Agilent Santa Clara, CA, USA) using a NUCLEODUR 100-5 CN-RP column (150 mm × 4.6 mm, 5 μm). Acetonitrile: 20 mM H_3_PO_4_ (16:84, *v*/*v*) was used as a mobile phase binary mixture, with an 0.8 mL min^−1^ flow rate at 30 °C, 20 µL of injection sample volume, and a detection wavelength of 220 nm. In the dissolution study, 50 mg of microparticles were suspended in 0.4 mL 1% agarose solution in a glass vial, and cooled to solidify the agarose, after which 800 μL of agarose was added and left to solidify, and 5 mL of phosphate buffer was added. At 4, 24, 48, 72, 96, and 168 h, 2 mL of buffer was collected and filtered through a 0.22 μm membrane filter. The remaining buffer was removed, the vials were washed with 0.5 mL of buffer to remove residue, and 5 mL of fresh buffer was added. In vitro experiments were performed at 5 °C in triplicate for each sample. The samples taken were analyzed by HPLC as above.

**Figure 1:**
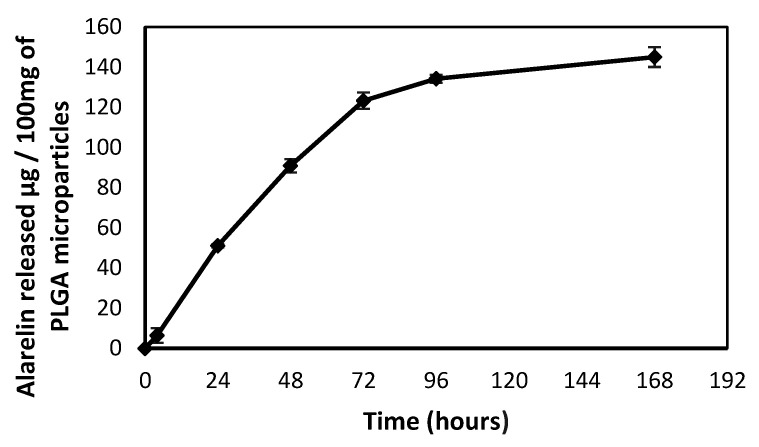
Release kinetics of Alarelin from PLGA microparticles.

Prepared PLGA microparticles contained 451.38 µg of Alarelin per 100 mg of sample (encapsulation efficiency of 43.32%). The release kinetics of prepared PLGA microparticles in agar gel for initial 168 h is shown in Figure 1. Within 72 h, Alarelin was released with almost regular increments per 24 h (51.1 µg/24 h; 90.90 µg/48 h; 123.31 µg/72 h). The sample was treated as a delivery system with 1.2 µg of Alarelin released/mg of PLGA microparticles/72 h.

#### 2.2.2. Treatments

Four groups of randomly selected sterlet males (10 per group) received a single intramuscular injection of one of four preparations suspended in saline solution (0.9% NaCl, Braun Melsungen AG, Melsungen, Germany) (Table 1).

### 2.3. Sample Collection

#### 2.3.1. Blood Collection

A heparinized 5 mL syringe with a heparinized 21-gauge needle was used to collect serial blood samples (1000 µL) by caudal venipuncture before injection (0 h), and at 24, 48, and 72 h post-injection. Blood samples were centrifuged at 4000× *g* for 10 min at 8 °C, and plasma was stored at −80 °C until analysis.

#### 2.3.2. Milt Collection

Milt was collected 24, 48, and 72 h post-treatment prior to blood collection. During the trial, fish were held at a constant temperature of 15 °C and natural illumination. Milt was collected by catheter from the urogenital duct into dry 50 mL plastic containers of known mass, avoiding contamination with feces or water. During collection, the male abdomen was gently massaged, allowing the complete release of milt from both Wolffian ducts. Milt from individual males was stored on ice at 4 °C for no longer than two hours during motility analysis.

### 2.4. Analysis of Samples

#### 2.4.1. 11-Ketotestosterone and Testosterone Analysis

The commercially available enzyme-linked immunosorbent assay kits were used to determine plasma levels of testosterone (T; KAPD1559; DIAsource ImmunoAssays SA, Louvain-la-Neuve, Belgium) and 11-ketotestosterone (11-KT; 582751; Cayman Chemical, MI, USA), according to the manufacturer’s instructions, with each standard and plasma sample run in duplicate. The intra-assay coefficients of variation for T and 11-KT, calculated from the sample duplicates, were less than 6% in all tests, and inter-assay coefficients of variation were less than 7% for T and 11-KT. The absorbance of all assays was read with a PlateReader AF2200 microplate reader (Eppendorf Czech and Slovakia s.r.o., Říčany u Prahy, Czech Republic).

#### 2.4.2. Sperm Quantitative Parameters

Spermatozoon concentration of each sample was estimated using a Burker cell hemocytometer (Meopta, Prerov, Czech Republic) at 200× magnification on an Olympus BX 50 phase-contrast microscope (Olympus Czech Group, Prague, Czech Republic). Each of the containers containing collected milt was individually weighted to 10 mg accuracy, and mass of milt was used as a proxy of milt volume. Sperm production was estimated by index of relative sperm production (RSP, 10^9^ spz/kg), computed as spermatozoon concentration multiplied by the volume of each sperm sample divided by the body weight of the corresponding male.

#### 2.4.3. Sperm Qualitative Parameters

After sperm collection, spermatozoon motility parameters were evaluated for each male. Motility of sperm samples was initiated in 10 mM Tris-HCl solution, pH 8.0, containing 0.125% Pluronic F-127 (catalogue number P2443, Sigma-Aldrich) to avoid sperm sticking to the glass slide. Motility was recorded at 50 frames per sec by optical negative phase-contrast microscopy, at ×10 magnification objective (PROISER, Madrid, Spain), and IDS digital camera (IDS Imaging Development Systems GmbH, Obersulm, Germany) for the first 100 s post-activation. Videos were analyzed to obtain kinetic data of spermatozoon motility with a 5 s interval starting at 10 s post-activation using the CASA plugin for ImageJ [20]. CASA analysis included the percent of motile cells, curvilinear velocity (VCL) in µm/s, and linearity (LIN) as ratio of velocity straight line to velocity average path (VSL/VAP). The cut-off for motile spermatozoa was set at VCL = 10 μm/s. Percent motility was determined at 10 s post-activation.

### 2.5. Statistical Analysis

#### 2.5.1. Spermiation Rate

A χ^2^ test was used to compare spermiation rate among the experimental groups.

#### 2.5.2. Quantitative Sperm Parameters and Androgen Concentrations

As data were not normally distributed and showed significant difference in dispersion values (Kolmogorov–Smirnov and Levene’s tests, respectively, *p* < 0.05), a nonparametric Kruskal–Wallis test was applied, followed by multiple comparisons of mean ranks for all groups. Tests were applied separately to compare groups at different times post-injection, and at the same time post-injection. The Mann–Whitney U-test was used for pairwise comparisons. An χ^2^ test was used to compare spermiation rate among the experimental groups.

#### 2.5.3. Sperm Qualitative Parameters

The sperm motility percentage, VCL, and LIN values for each combination of fish/experimental group/sampling time were extracted from the CASA dataset. Mean data of individual fish for each experimental condition were used to plot trend lines of VCL at 10–100 s post-activation time. Quadratic polynomial regression was selected for visualization of parameter trends. Before analysis, motility percentage and VCL data were tested for normality and homogeneity of variance by Kolmogorov–Smirnov and Levene’s tests, respectively. All studied parameters were normally distributed and exhibited similar dispersion values. Data were analyzed by one-way ANOVA followed by Tukey’s test.

Data of motility rate were transformed by the BOX-COX procedure before statistical analysis to obtain equality of variance among groups and analyzed first by two-way ANOVA with treatment as three levels (CP, PLG35, and PLG200), and post-injection time of three levels (24, 48, and 72 h). Tukey’s test was used to assess differences among mean values of spermatozoon motility percentage in experimental groups.

Statistical analysis and graph plotting was performed using Statistica v. 13.5.0.17. (TIBCO Software Inc., Palo Alto, CA, USA).

## 3. Results

### 3.1. Androgen Concentrations

#### 3.1.1. Testosterone Concentrations

**Figure 2:**
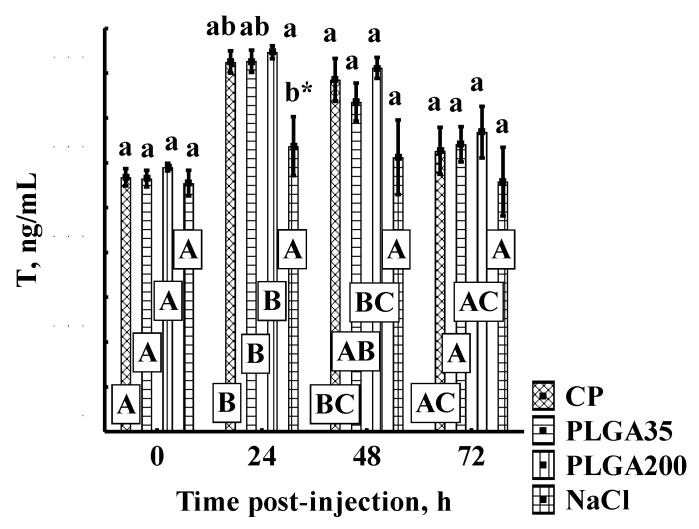
Testosterone concentration after hormonal treatments in sterlet. Significant differences among groups at a sampling point are indicated by lower case letters (*p* < 0.05). Significant differences within an experimental group at a different post-injection time are indicated by upper case letters (multiple comparisons of mean ranks for all groups, *p* < 0.05). *—indicates a significant pairwise difference between treatment by NaCl (control, dose 1 mL (0.9%NaCl)/kg) and each experimental group at 24 h post-injection (Mann–Whitney U-test, *p* < 0.05). Experimental treatments: CP—carp pituitary extract, dose 4 mg/kg; PLGA35—Alarelin, dose 35 µg/kg; PLGA200—Alarelin, dose 200 µg/kg.

No differences in T concentration were observed among experimental groups at 0 h, 48 h, and 72 h, with the only intergroup differences between the PLGA and control groups observed at 24 h post-treatment. Administration of hormone treatment induced a significant increase in T values at 24 h compared to initial values, and declined thereafter (Figure 2).

#### 3.1.2. 11-KT Concentrations

**Figure 3:**
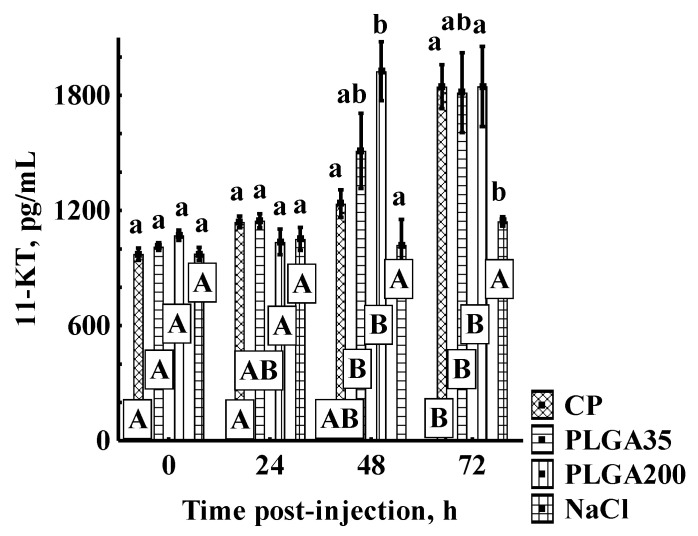
11-KT concentration with and without hormone treatment in sterlet. Significant differences among groups at a sampling point are indicated by lower case letters (*p* < 0.05). Significant differences within an experimental group are indicated by upper case letters (*p* < 0.05). Experimental treatments: CP—carp pituitary extract, dose 4 mg/kg; PLGA35—Alarelin, dose 35 µg/kg; PLGA200—Alarelin, dose 200 µg/kg; NaCl—control, dose 1 mL (0.9% NaCl)/kg.

No differences in 11-KT concentration were observed among experimental groups at 0 h and 24 h. No significant differences were detected between PLGA35 and PLGA200 groups at any sampling point. Administration of PLGA35 did not stimulate values of 11-KT significantly different from those detected in the control or CP group at any sampling point. PLGA200 induced higher values than NaCl at 48 h and 72 h and higher levels than CP at 48 h post-treatment. CP was associated with higher values than the control only at 72 h post-treatment (Figure 3).

Application of PLGA induced 11-KT values at 48 h and 72 h post-treatment significantly higher than initial values. Groups receiving CP showed a significant increase from initial values 72 h post-treatment (Figure 3).

### 3.2. Effect of Hormone Treatment on Sperm Production

#### 3.2.1. Spermiation Rate

Spermiation was observed in six males in the CP group at 24 and 48 h post-injection, nine males in the PLGA35 group at 24, 48, and 72 h, and eight males in the PLGA200 group at 24, 48, and 72 h. No sperm was obtained from fish injected with saline solution. No significant differences were found among hormone-treated groups in the proportion of fish showing spermiation, and all treated groups differed significantly from the control group (*p* < 0.05).

#### 3.2.2. Sperm Quantitative Parameters

##### Milt Volume

**Figure 4:**
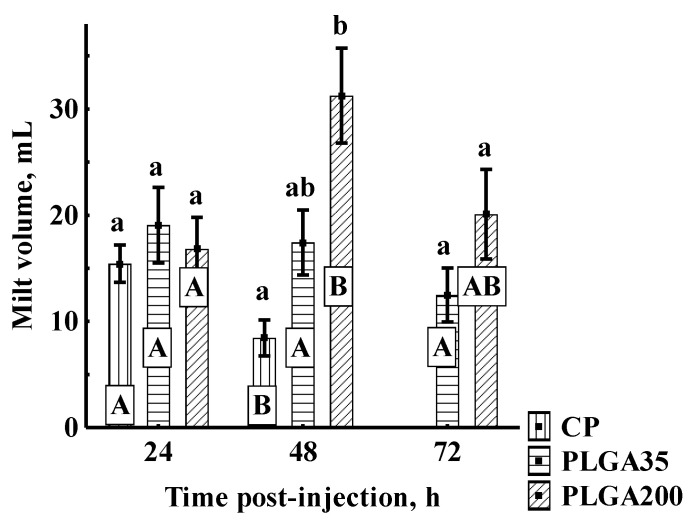
Milt volume after hormone treatment. Significant differences among groups at a sampling point are indicated by lowercase letters (*p* < 0.05). Significant differences within an experimental group are indicated by uppercase letters (*p* < 0.05). Experimental treatments: CP—carp pituitary extract, dose 4 mg/kg; PLGA35—Alarelin, dose 35 µg/kg; PLGA200—Alarelin, dose 200 µg/kg.

No significant differences in milt volume were observed among experimental groups 24 h post-treatment. At 48 h post-treatment, the PLGA200 group produced a significantly higher volume of sperm than seen with CP. A significant decrease in milt volume was observed in the group stimulated by CP at the second sampling time (48 h), with no spermiation detected at 72 h. Administration of PLGA treatments led to consistent volume throughout the trial period with no differences within or among groups (Figure 4).

##### Spermatozoon Concentration

**Figure 5:**
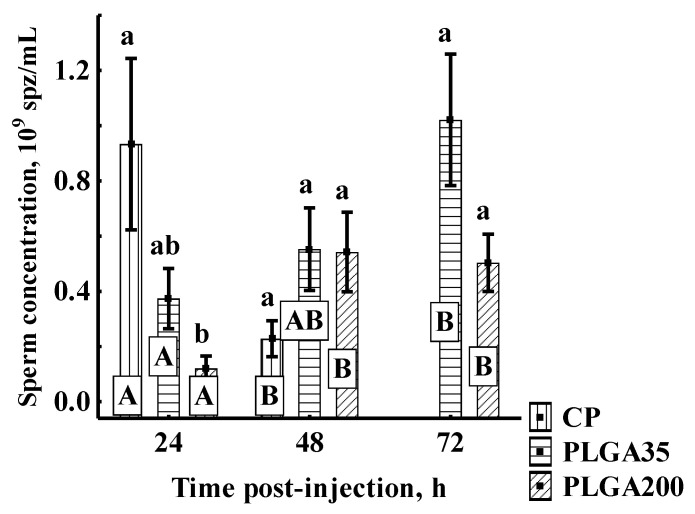
Spermatozoon concentration after hormone treatment. Significant differences among groups at a sampling point are indicated by lowercase letters (*p* < 0.05). Significant differences within an experimental group are indicated by uppercase letters (*p* < 0.05). Experimental treatments: CP—carp pituitary extract, dose 4 mg/kg; PLGA35—Alarelin, dose 35 µg/kg; PLGA200—Alarelin, dose 200 µg/kg.

CP initially induced high spermatozoon concentration 24 h post-treatment followed by a significant decrease at 48 h. Concentration in the PLGA35 group reached highest levels at 72 h, but did not differ from other groups at any sampling point. The PLGA200 group exhibited lower initial sperm concentration than with CP, but did not differ from PLGA35. Significantly higher values for PLGA200 were observed at 48 h and 72 h post-treatment compared to values at 0 h, although not differing at 48 and 72 h from other groups (Figure 5).

##### Relative Sperm Production

**Figure 6:**
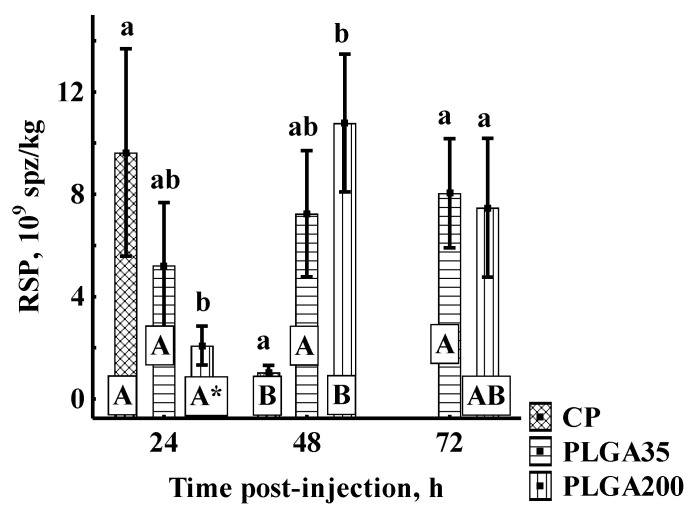
Relative sperm production (RSP) following hormone treatment. Significant differences among groups at a sampling point are indicated by lowercase letters (*p* < 0.05). Significant differences within an experimental group are indicated by uppercase letters (*p* < 0.05). *—indicates a significant pairwise difference between groups treated by PLGA 200 at 24 h in comparison to groups treated by PLGA200 at 48 h and 72 h post-injection (Mann–Whitney U-test, *p* < 0.05). Experimental treatments: CP—carp pituitary extract, dose 4 mg/kg; PLGA35—Alarelin, dose 35 µg/kg; PLGA200—Alarelin, dose 200 µg/kg.

Similar to the trend in spermatozoon concentration, the administration of CP led to high RSP at 24 h post-treatment followed by a significant decline at 48 h, significantly differing at all points from PLGA200 but not from PLGA35. The PLGA35 group showed consistent RSP throughout the experiment, and did not differ from other groups at any sampling point. Application of PLGA200 was associated with lower initial RSP with significant increase at 48 h and 72 h (Figure 6).

#### 3.2.3. Sperm Qualitative Parameters

##### Spermatozoon Curvilinear Velocity and Linearity

**Figure 7:**
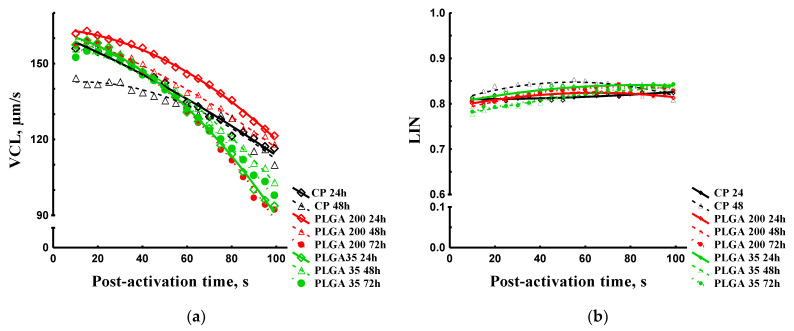
Kinematic parameters of sterlet spermatozoa obtained after hormone treatments. (**a**) VCL dynamics post-activation. (**b**) LIN dynamics post-activation. Data are presented as mean (dots) and quadric polynomial regression lines. Abbreviations: CP 24 h, CP 48 h-treatment by carp pituitary extract, dose 4 mg/kg, samples collected at 24 and 48 h post-injection respectively; PLGA 200 24 h, PLGA 200 48 h, PLGA 200 72 h—treatment by Alarelin, dose 200 µg/kg, samples collected at 24, 48, and 72 h post-injection respectively; PLGA 35 24 h, PLGA 35 48 h, PLGA 35 72 h—treatment by Alarelin, dose 35 µg/kg, samples collected at 24, 48, and 72 h post-injection respectively.

After spermatozoon motility activation, VCL decreased in all experimental groups (Figure 7a) with no differences among groups. The dynamics of mean LIN values in experimental groups did not allow selection of points of interest, as values were similar, and no significant differences were found.

##### Spermatozoon Motility Rate

**Figure 8:**
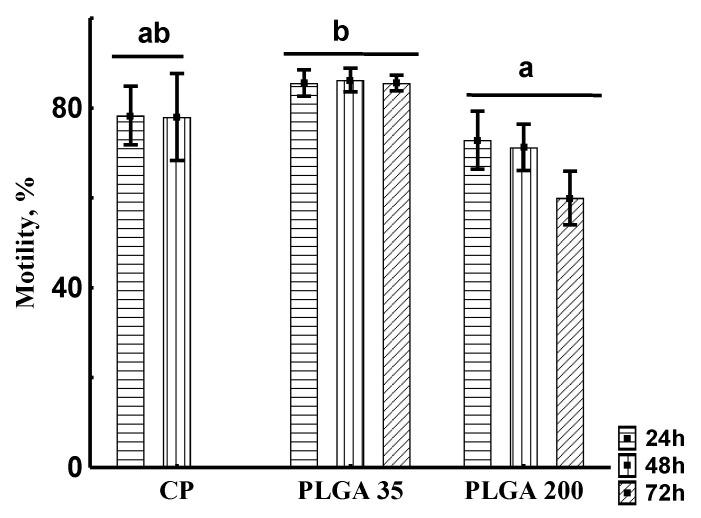
Sperm motility percentage (Motility, 10 s post-activation) of sterlet sperm samples obtained after hormonal treatments at different post-injection times (24, 48, and 72 h). Horizontal Lines indicate no significant f factor “post-injection time” (two-way ANOVA, *p* = 0.989). Different letters indicate significant differences among treatments by different hormones, combined by the factor “post-injection time” (two-way ANOVA, *p* < 0.001; Tukey’s post-hoc test, *p* < 0.05). Experimental treatments: CP—carp pituitary extract, dose 4 mg/kg; PLGA35—Alarelin, dose 35 µg/kg; PLGA200—Alarelin, dose 200 µg/kg.

Two-way ANOVA applied to motility rate revealed a significant effect of treatment (*p* < 0.001). In contrast, effects of time post-injection and the interaction of these two factors were not significant (*p* = 0.989 and *p* = 0.751, respectively). Tukey’s test was used to compare mean values of spermatozoon motility rate in experimental groups without consideration of time post-injection. Motility rate was significantly lower in the PLGA200 group than in PLGA35, while no differences were found between CP and the PLGA groups (Figure 8).

## 4. Discussion

Hormone treatment is a prerequisite for successful spermiation in cultured sterlet males [19]. In the current study, a PLGA microparticle system with continuous Alarelin release significantly prolonged the spermiation period and increased the number of expressible spermatozoa, as compared to standard treatment by carp pituitary suspension.

The spermiation-stimulating effect of CP suspension has been known for decades, and it is widely used in sturgeon aquaculture [9]. The CP directly stimulates testicular steroidogenesis and does not rely on endogenous LH stores in the pituitary [5].

Administration of CP in our trial stimulated initiation of spermiation with maximum RSP 24 h post-treatment followed by a significant decrease at 48 h and no expressible sperm at 72 h post-treatment. A time curve of spermiation decline after CP treatment similar to our findings has been previously reported in sterlet [21] and in paddlefish [22]. The spermiation-inducing effect of gonadotropin preparations is usually more rapid and shorter-lasting than GnRHa treatment [4,22]. Analogues of GnRH act at a higher level of the hypothalamic-pituitary-gonad axis, and induce secretion of endogenous LH and possibly other factors that may be involved in the regulation of spermiation [23]. In sterlet, the administration of GnRHa alone dissolved in saline solution has not been shown to initiate satisfactory sperm production [19,24]. The low efficacy of a single GnRHa injection in inducing spermiation in sterlet is probably a combined result of the short residence time of GnRHa in circulation [25] and dopamine inhibition of LH secretion [19,24]. Co-administration of GnRHa with a dopamine antagonist led to a significant improvement of spermiation parameters compared to GnRHa alone [24]. Combined treatment of GnRHa and the dopamine antagonist used by Alavi et al. [4] stimulated maximal sperm production 48 h post treatment, followed by a significant decline at 72 h post treatment.

One of the advantages of the GnRHa peptide is its low molecular weight and efficacy in μg doses, enabling its incorporation into delivery systems with release over a prolonged period [8]. The controlled delivery systems of GnRHa have proven to be effective stimulators of spermiation in several marine species [11]. Mentioned results with marine species are in agreement with our observation in sterlet, where PLGA microparticle system with continuous Alarelin release enabled production of high-quality sperm over 72 h with no differences between PLGA35 and CP at 24 and 48 h post-treatment. Interestingly the treatment PLGA200 (200 µg kg^−1^) was associated with a significantly lower RSP than the CP group at 24 h, but no difference was found in milt volume.

In contrast to our results, unsatisfactory spermiation using slow-release EVAc was reported by Alavi et al. [4]. This may have resulted from the release kinetics of Alarelin from PLGA microparticles compared to EVAc implants. Release of Alarelin from PLGA microparticles is characterized by an immediate initial burst and a sustained or continuous decline until depletion of the microspheres (Figure 1). Significant portion of Alarelin incorporated in our delivery systems was released within 72 h post-injection. The Alarelin at 35 µg kg^−1^ induced adequate spermiation at all sampling points and outperformed the 200 µg kg^−1^ treatment in spermatozoon motility. Experiments in other fish species have confirmed that unnecessarily high doses of GnRHa can negatively influence final gamete maturation [26,27]. No differences were detected between sperm samples obtained after treatment by CP and PLGA delivery systems in term of velocity or linearity parameters.

Final sperm maturation is a crucial component of the life cycle of fish, being under the control of the LH stimulating production of sex steroids [28]. Androgens T and 11-KT are the predominant sex steroids in male teleost fish [29]. In the current study, a positive correlation between T and 11-KT was observed, with all the hormone treatments significantly increasing T and 11-KT values, a decline in T at the end of the trial, and remaining high values of 11-KT. This observation is consistent with the fact that T acts as a precursor of 11-KT, with 11-KT playing an important role in the initiation of spermiation [30].

The efficacy of the PLGA microparticle delivery system compared to acute application of GnRHa in saline solution demonstrates the ability of sustained Alarelin release to stimulate satisfactory spermiation results, despite the assumed dopamine inhibition of LH secretion in sterlet males [19,24], and induce favorable results both quantitively and qualitatively. The elimination of a dopamine antagonist in spermiation treatment of sterlet is desirable, considering its long half-life and wide range of potential side effects.

## 5. Conclusions

A PLGA microparticle system with sustained release of Alarelin at 35 µg kg^−1^ effectively induces spermiation in sterlet, significantly outperforming CP in prolonged stimulation of spermiation.

## Figures and Tables

**Table 1:** Experimental groups of sterlet males.

Treatment	Fish Weight (kg)	Dose
0.9% NaCl	1.57 ± 0.23	1 mL/kg
Carp pituitary extract	1.59 ± 0.22	4 mg/kg
PLGA35 ^1^	1.59 ± 0.25	35 µg/kg Alarelin
PLGA200 ^1^	1.58 ± 0.27	200 µg/kg Alarelin

^1^ PLGA microparticle delivery system with sustained Alarelin release.

## Data Availability

The data presented in this study are available on request from the corresponding author.
